# Ambulatory blood pressure monitoring using telemedicine: proof-of-concept cohort and failure modes and effects analyses

**DOI:** 10.12688/wellcomeopenres.17537.2

**Published:** 2022-04-06

**Authors:** Laura C. Armitage, Beth K. Lawson, Cristian Roman, Beth Thompson, Christopher Biggs, Heather Rutter, Martin Lewis-Jones, Jody Ede, Lionel Tarassenko, Andrew Farmer, Peter Watkinson

**Affiliations:** 1 Nuffield Department of Primary Care Health Sciences, University of Oxford, Oxford, UK; 2 Institute of Biomedical Engineering, Department of Engineering Science, University of Oxford, Oxford, UK; 3 Oxford University Hospital NHS Foundation Trust, Oxford, UK; 4 Nuffield Department of Clinical Neurosciences, University of Oxford, Oxford, UK; 5 Heart Voices, British Heart Foundation, London, UK

**Keywords:** Hypertension, Telemedicine, Screening, Cardiovascular Disease, Blood Pressure Monitoring, Ambulatory Blood Pressure Monitoring

## Abstract

**Background: **The COVID-19 pandemic has accelerated adoption of remote consulting in healthcare. Despite opportunities posed by telemedicine, most hypertension services in Europe have suspended ambulatory blood pressure monitoring (ABPM).

**Methods: **We examined the process and performance of remotely delivered ABPM using two methodologies: firstly, a Failure Modes and Effects Analysis (FMEA) and secondly, a quantitative analysis comparing ABPM data from a subgroup of 65 participants of the Screening for Hypertension in the INpatient Environment (SHINE) diagnostic accuracy study. The FMEA was performed over seven sessions from February to March 2021, with a multidisciplinary team comprising a patient representative, a research coordinator with technical expertise and four research clinicians.

**Results: **The FMEA identified a single high-risk step in the remote ABPM process. This was cleaning of monitoring equipment in the context of the COVID-19 pandemic, unrelated to the remote setting.

A total of 14 participants were scheduled for face-to-face ABPM appointments, before the UK March 2020 COVID-19 lockdown; 62 were scheduled for remote ABPM appointments since emergence of the COVID-19 pandemic between November 2020 and August 2021. A total of 65 (88%) participants completed ABPMs; all obtained sufficient successful measurements for interpretation. For the 10 participants who completed face-to-face ABPM, there were 402 attempted ABPM measurements and 361 (89%) were successful. For the 55 participants who completed remote ABPM, there were 2516 attempted measurements and 2214 (88%) were successful. There was no significant difference in the mean per-participant error rate between face-to-face (0.100, SD 0.009) and remote (0.143, SD 0.132) cohorts (95% CI for the difference -0.125 to 0.045 and two-tailed P-value 0.353).

**Conclusions: **We have demonstrated that ABPM can be safely and appropriately provided in the community remotely and without face-to-face contact, using video technology for remote fitting appointments, alongside courier services for delivery of equipment to participants.

## Introduction

The World Health Organization states “A good health system delivers quality services to all people, when and where they need them” ^1^. In 2020, the outbreak of the COVID-19 pandemic accelerated a move to remote consultations in UK healthcare ^2^. Whilst this ensured a number of services continued to be accessed by a proportion of the population when and where they needed them, this was not universal. Some services, such as ambulatory blood pressure monitoring (ABPM), became inaccessible to patients and participants in clinical and research settings ^3^.

ABPM was first introduced to regular clinical use in the late 1980s ^4^. Since then, 24-hour ABPM has become the gold standard method for assessing for hypertension in the UK and Europe ^5^. However, the European Society of Hypertension Coronavirus Disease 19 Task Force reported that 57% of hypertension excellence centres in Europe ceased delivering 24-hour ABPM during the COVID-19 pandemic ^3^. Where ABPM has continued, provision is often limited to selected clinical scenarios such as pregnancy or following a hypertensive emergency ^3^. The major barrier to service continuation has been the face-to-face contact required between healthcare professional and patient. Standard practice traditionally requires face-to-face appointments to complete safety screening checks, fit the monitor, and remove it 24 hours later for data download with interpretation ^6^. Whilst home blood pressure monitoring has been utilised for diagnostic and monitoring purposes during the COVID-19 pandemic, it is inferior to ABPM in that it does not provide information on a person’s blood pressure during activities of daily living, sleep, or 24-hour variability in blood pressure ^7^. Blood pressure measurements obtained from ABPM are also a better predictor of hypertension-mediated organ disease ^8^. As the effects of the COVID-19 pandemic extend with time, new ways of delivering services, including ABPM, must be considered and evaluated to continue delivering gold-standard diagnostics, maintain standards of care, and offer resilient healthcare services accessible to patients when and where they are needed.

In 2019, we began recruiting NHS patients to the Screening for Hypertension in the INpatient Environment (SHINE) study at the Oxford University Hospitals NHS Foundation Trust, UK ^9^. In March 2020, all clinical research that was not essential to delivery of care or concerning COVID-19 was suspended. Upon resumption of recruitment in September 2020, we had amended the SHINE study protocol ^9^ to minimise face-to-face contact between participants and clinical researchers, reducing risk of transmission of COVID-19. We designed a procedure for delivering ABPM remotely to participants, whilst still adhering to the British and Irish Heart Society Standard Operating Procedure (SOP) for the performance of ABPM ^6^ and their resources for clinical services providing ABPM ^10^.

We identified Failure Modes and Effects Analysis (FMEA) as an appropriate methodological approach for a detailed analysis of the potential risks and possibilities for failure that might arise from adapting ABPM to a remote service. FMEA provides a framework for the systematic, in-depth evaluation of a specific process, to identify where and how the process may fail and assess the potential effect of failures ^11^. Once potential failure points are identified, preventive measures are prioritised according to the likelihood of the failure, risks and effects ^12^. A recent systematic review highlighted the broad and increasing use of FMEA in healthcare, to evaluate a range of services including drug administration and delivery, blood transfusion, treatment of sepsis and surgical procedures ^13^. The authors concluded that FMEA can proactively reduce errors in medicine and improve quality of care, particularly in the context of increasing sophistication and complexity of medical interventions, equipment and related processes ^13^.

The objective of this study was to examine the process and performance of ABPM when delivered remotely, using FMEA and a quantitative analysis that compared ambulatory blood pressure data from participants receiving remote ABPM appointments, versus ambulatory blood pressure data from participants receiving face-to-face ABPM appointments.

## Methods

### Study registration

The SHINE Study protocol was registered with the ISCTRN Registry (Identification number ISRCTN80586284, date 20 August 2019).

### Study design and setting

Firstly, we evaluated the process of remote ABPM, its potential risks, failure points and the impacts of these using FMEA. A multi-disciplinary FMEA panel was assembled comprising a patient and public representative, a research coordinator with technical expertise, a General Practitioner, a physiotherapist and two clinical research nurses. An initial training and introductory session in FMEA was conducted for the panel, followed by six weekly sessions between February and March 2021. During these six sessions we systematically worked through the process of an episode of ABPM, using an FMEA framework. First, the process of interest was identified (remote performance of ABPM), followed by the main steps (e.g. scheduling the 24-hour ABPM episode with the participant) and then sub-steps involved in the process (e.g. phoning the participant, confirming eligibility, agreeing a date for monitoring, configuring the monitor and scheduling courier delivery). These steps and sub-steps were identified using the participant and researcher guides that were developed for the remote delivery of ABPM. These study guides were developed with reference to the British and Irish Hypertension Society’s (BIHS) Standard Operating Procedure for ABPM ^6^, the BIHS Clinic Checklist and Educational Resource Video for ABPM ^10^, and the UK NICE Guidelines for diagnosing and managing hypertension ^5^. The team identified the potential failure modes (ways in which a failure could occur), failure causes (what might lead to a failure occurring) and the failure effects (consequences) for each sub-step. Scores were then assigned to each of the failure modes as described further under the ‘Measures’ sub-heading below.

Secondly, we evaluated the performance of remote ABPM by analysing the proportion of successful 24-hour ABPM monitoring episodes prior to the onset of the UK COVID-19 epidemic (before which time the procedure was delivered by face-to-face appointments) and since the UK COVID-19 epidemic (since which time the procedure has been delivered using telemedicine). We also investigated the rate of successful ABPM measurements, per 24-hour period in the face-to-face versus remote ABPM groups.

### Participants

Participants included in the analysis of the *performance* of remote ABPM were a subgroup of those enrolled on the SHINE diagnostic accuracy study who had, following discharge from hospital (index admission), worn a 24-hour blood pressure monitor in accordance with the SHINE study protocol ^9^. All participants gave written informed consent for their participation in the study. The subgroup consisted of two cohorts, the first cohort being all participants who attended fitting and removal ABPM appointments face-to-face prior to the UK coronavirus epidemic in 2020, the second cohort being all participants who undertook fitting and removal of the ABPM through remote appointments using telemedicine, from November 2020 to August 2021. The full inclusion and exclusion criteria for the SHINE study have been published elsewhere ^9^, but in short, included adult patients aged 18–80, admitted to Oxford University Hospitals NHS Foundation Trust, UK for a minimum of 24-hours and no previous or existing diagnosis of, or prescription for, hypertension or atrial fibrillation.

### Intervention

The intervention evaluated in this study was the remote performance of 24-hour ABPM, using a courier service to deliver and retrieve the monitoring equipment, and telemedicine to complete fitting and removal appointments with participants. The comparator was 24-hour ABPM with traditional face-to-face consulting at a primary care health centre to complete fitting and removal of the ABPM. Participants in both the face-to-face and remote ABPM groups were provided with a Mobil-o-graph NG 24hr BP Monitor System (IEM Healthcare, Stolberg, Germany), serviced and calibrated according to the manufacturer’s recommendations.

**Figure 1.**
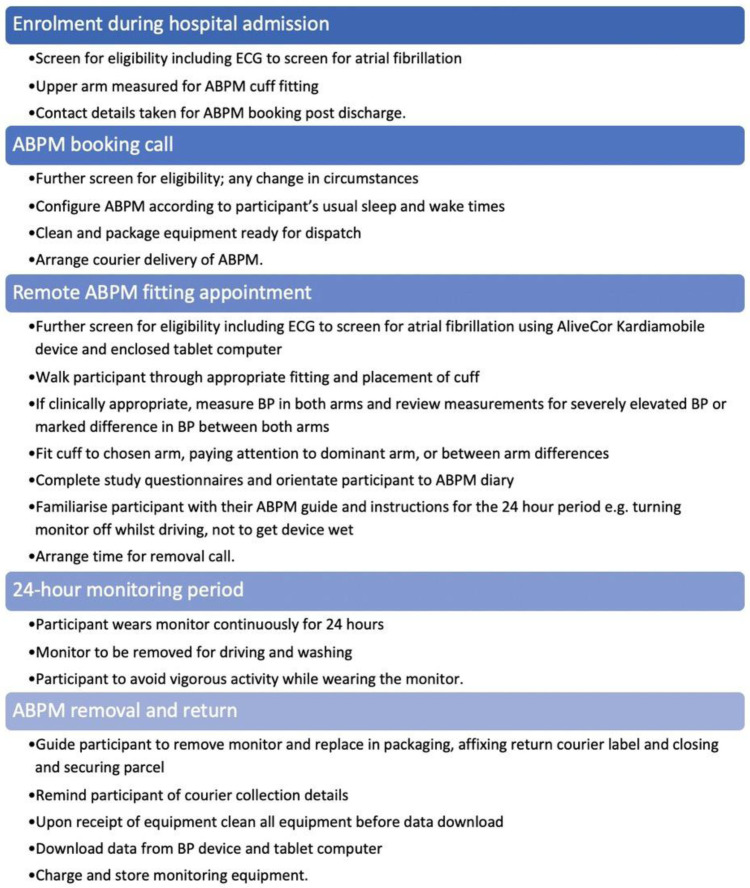
Summary of the remote ambulatory blood pressure monitoring (ABPM) process.

Both the face-to-face and remote 24-hour ABPM processes were based on the ABPM process outlined in the BIHS SOP for Ambulatory Blood Pressure Monitoring ^6^, with close attention to maintaining standard safety checks for atrial fibrillation, other contraindications for ABPM and severely elevated blood pressure. An overview of the process for performing remote ABPM is presented in Figure 1, and details regarding the safety checks are presented in Table 1. The process involved initial screening for eligibility and suitability at enrolment during the participant’s index hospital admission as per the SHINE protocol ^9^. Once participants were enrolled, their upper arms were measured to assign the correct-sized ABPM cuff to be dispatched with the ABPM for the remote fitting appointment. After discharge from hospital, participants were contacted by telephone to arrange the remote-fitting appointment, and to collect details about their sleep and wake patterns for tailored configuration of monitor settings. The validated and calibrated monitor was then couriered to the participant, along with an AliveCor KardiaMobile ECG device (AliveCor Inc, Mountain View, CA), and a tablet computer with a SIM card installed for 4G internet connectivity. The tablet computer had the secure video-calling Nye Health App (Nye Health Ltd, Oxford, UK) and the ECG partner application Kardia pre-installed (both applications downloaded from the Google Play app store https://play.google.com/store/apps and regularly updated to the latest application versions throughout the study period). Video appointments for ABPM fittings were completed using the Nye Health App. During the appointment, the AliveCor KardiaMobile ECG device recorded data to the Kardia app on the tablet computer, with the app generating automated real-time ECG interpretation. This enabled clinical research staff to screen for atrial fibrillation, that would exclude participants from being eligible to proceed with ABPM. Following the ECG recording, the participant was walked through the checking of their blood pressure using the device in both arms, before being shown how to fit the monitor to the most appropriate arm and proceeding with the 24-hour monitoring. At least twenty-four hours following the fitting appointment, the participant was phoned to confirm removal of the ABPM and complete removal and return steps.

**Table 1.** Safety checks performed during remote configuration, fitting and removal of a 24-hour ABPM.

Safety check ►	Screen for severely elevated blood pressure			Screen for contraindications to ABPM on one or more limbs			Screen for arrhythmias		
Timepoint in study ▼	Included	Procedure	Definition	Included	Procedure	Definition	Included	Procedure	Definition
**At enrolment**	Yes	• Average 24-hour blood pressure measured by screening algorithm **and** • Enrolment blood pressure performed by clinical research nurse.	• Average 24-hour BP ≥180mmHg systolic or ≥120 mmHg diastolic • Enrolment blood pressure ≥180 mmHg systolic or ≥120mmHg diastolic	Yes	• Review of hospital medical records and, if available, primary care summary care record. • Verbal screening with participant		Yes	• 6-lead ECG using AliveCor KardiaMobile 6L ECG device with automated screening for AF and instant report • Clinician review of ECG and automated report for accuracy	• Automated report showing atrial fibrillation • Absence of p waves and irregularly irregular QRS complex on clinician review
**ABPM** ** booking call**	No	• n/a	• n/a	Yes	• Verbal screening with participant		No	• n/a	
**ABPM fitting** ** appointment**	Yes	• Blood pressure checked in both arms in accordance with British and Irish Hypertension Society SOP for ABPM fitting.	• Blood pressure measurement ≥180/110mmHg • *Between arm BP difference ≥20mmHg systolic or ≥10mmHg diastolic	Yes	• Verbal screening with participant		Yes	• Single-lead ECG using AliveCor KardiaMobile ECG device with automated screening for AF and instant report	• Automated report showing atrial fibrillation
**ABPM data** ** download**	Yes	• Review of mean daytime blood pressure	• Mean daytime blood pressure ≥160/105 mmHg	n/a	n/a		Yes	• Clinician review of ECG and automated report for accuracy	• Absence of p waves and irregularly irregular QRS complex

*Denotes screen for significant inter-arm blood pressure difference. *n/a = not applicable, SOP = Standard Operating Procedure, AF = Atrial fibrillation, ECG = electrocardiogram*

### Measures

***Failure modes and effects analysis.*** The failure modes assigned to each of the sub-steps identified were assigned three initial scores on a scale of 1–10, based on likelihood of failure (1 being very unlikely and 10 being very likely), likelihood the failure would go undetected (1 being very unlikely and 10 being very likely) and severity of the effects (1 being minor or only a slight annoyance with 10 being very severe and causing harm to a patient, researcher or the study). A key for the scoring is shown in Table 2. These three scores were then multiplied by one another to calculate risk priority numbers (RPN). Those sub-steps with the highest RPN were deemed to be priority steps for identifying remedial actions to be proactively addressed to prevent, detect and mitigate failure of the remote ABPM process.

**Table 2.** Scoring key for the failure modes associated with each sub-step in the ABPM process ^11, 14^.

Rating	Likelihood of occurrence	Likelihood of detection	Severity of risk
**1**	Remote – no known recurrence	Certain – error will always be detected	Slight annoyance only – no injury to participant or research staff and no impact on study
**2**	Rare – yearly	Very high probability of detection	Slight danger – but with no injury to participant or research staff or slight impact on study
**3, 4**	Occasional – quarterly	High probability of detection	Low to moderate danger – very minor or no injury to the participant or research staff and minimal impact on study
**5, 6**	Moderately frequent - monthly	Moderate chance of detection	Moderate danger – minor or no injury to participant or research staff, moderate impact on study
**7, 8**	Very frequent – weekly	Low chance of detection	Dangerous – minor or moderate injury to the participant or research staff and/or marked impact on study.
**9**	Inevitable	Remote chance of detection	Very dangerous – may result in major injury to participant or research staff and/or major impact on study.
**10**	Certain – daily	No chance of detection	Extremely dangerous – may cause death to participant.

***Analysis of the performance of remote ambulatory blood pressure monitoring.*** We planned *a priori* to assess the proportion of successful 24-hour ABPM episodes and ABPM measurements within that 24-hour period, in each participant cohort. A 24-hour ABPM episode was deemed successful and suitable for diagnostic interpretation if ≥14 measurements were obtained during waking hours, as defined by the UK NICE Guidelines for the diagnosis and management of hypertension ^5^. We also assessed the number of attempted BP measurements per participant during the period of monitor wear, and the number of failed BP measurements per participant during the same period (denoted by a time-stamped error message on the ABPM report; the list of error messages returned is detailed in Table 3). From these we calculated the error rate for each participant as the number of failed BP measurements divided by the number of attempted BP measurements. The mean error rate was then calculated for the face-to-face ABPM cohort and the remote ABPM cohort.

**Table 3.** Error messages analysed during 24-hour ambulatory blood pressure monitoring.

Measurement comments contributing to calculation of error rate in ABPM episodes	Measurement comments not regarded erroneous and therefore not included in calculation of error rate in ABPM episodes
Pressure increased during deflation. Movement?	Start of a manual measurement
Difference between the systolic and diastolic value is too small	Device was switched off
Movement artefact	Event button
The heart rate was outside the defined range	The day/night button was not pressed during the set time frame
Exceeded measurement limit	Restarted during a 24h profile
Measurement aborted by the user	
Difference between the systolic and diastolic value is too small	
Can not determine the blood pressure	
[Druck zu groß.] (translates to pressure too great)	
Cuff inflation was too fast. Is there a kink?	
Undefined error	
Pressure cannot be increased fast enough. Leakage?	

The sample size for the face-to-face ABPM cohort was not within our control, owing to the short time during which we were able to recruit and follow up participants prior to suspension of research activity during the first wave of the UK COVID-19 epidemic. Whilst the sample size was not powered to assess for a statistically significant difference, we performed a t-test (GraphPad Prism, San Diego, USA) to investigate for a significant difference between the mean error rate for the two participant cohorts.

### Ethical review and participant consent

Ethical approval for the SHINE study has been provided by the National Health Service Health Research Authority South Central—Oxford B Research Ethics Committee (19/SC/0026).

All participants in this study gave written consent for their involvement in the study, and for the publication of non-identifiable reports of results and scientific manuscripts, available in the public domain.

## Results

### Failure modes and effects analysis

***Identifying key steps in the process and potential failure modes.*** The FMEA panel identified four key stages in the process for remote ABPM which were the remote fitting appointment, the 24-hour monitoring period, the remote removal appointment and equipment return and data download. Each stage was divided into a total of 14 steps and then 42 sub-steps. Potential failure modes, causes and effects were identified for each of these 42 sub-steps. Several of the sub-steps were potentially at risk of multiple failure modes but all of which would result in the same effects, and the same likelihood of detection and risk severity. We therefore assigned the scores for likelihood of occurrence, likelihood of detection and severity of risk to the groups of failure modes that belonged to each sub-step. Each sub-step was therefore assigned a RPN. Those with the highest ranking RPNs are reported in Table 4.

**Table 4.** Failure Modes and Effects Analysis results for low-moderate and high-moderate risk sub-steps in remote ABPM.

Step of remote ABPM	Sub-step	Failure mode	Failure cause	Failure effects	Likelihood of occurrence	Likelihood of detection	Severity	Risk priority number
**Contact participant ** **to book 24-hour** ** ABPM, programme ** **monitor and ** **schedule courier ** **delivery**	Reconfirm eligibility at booking call	Patient proceeds to ABPM but would not have been eligible due to change in circumstance since hospital admission	Eligibility not checked during this phone call	Inappropriate use of study resources, inconvenience to the participant, inaccurate data if error not detected, non-usable/destroyed data if error detected.	2	4	4	32
	Configure the monitor to record the participant’s blood pressure twice hourly in waking hours and once hourly in sleeping hours	Measurements performed at incorrect frequency	Incorrect sleep and wake times entered or incorrect frequency selected when configuring monitor	Insufficient waking time measurements to assess for the presence of daytime hypertension in keeping with UK guidance ^5^. Sleep disturbance to participant.	2	2	6	24
	Arrange courier	ABPM and associated equipment do not arrive with participant	Incorrect address, change in participant schedule since booking phone call, too great a time between booking call and delivery date, courier error, theft of equipment.	Financial loss to study owing to loss of equipment. Human resource in rearranging and reordering equipment, delay in follow up and data collection for participant and if delay major then breach of follow up window permitted by protocol and participant withdrawal	4	1	3	12
**Fit the ABPM to the ** **chosen arm for the** ** 24-hour monitoring**	Explain to the participant where the ABPM tubing should lie	Tubing placed incorrectly	Participant confused by instructions, participant isn’t instructed how to thread the tubing through clothing down the back	Multiple attempts at repeat measurements, by the automated BP device, multiple erroneous/failed measurements	6	1	2	12
**Participant ** **commences and ** **completes 24-hour ** **ABPM**	Participant advised and aware not to exercise any more vigorously than brisk walking during the monitoring period	Participant may wear monitor during vigorous activity if ambiguity/subjectivity in its interpretation; participant may remove the monitor during brisk activity and forget to replace it or turn it back on after exercising.	Participant unable to avoid vigorous activity due to lifestyle, instruction regarding avoiding vigorous activity not given to participant	Potential for elevated or failed BP measurements if monitor attempts to take measurements during vigorous exercise; loss of a period of measurements if participant forgets to resume monitoring after exercise.	2	2	3	12
**Clean equipment ** **upon its return** ** to the research** ** centre, prior to ** **data download**	n/a	Equipment not cleaned after unpacking	Equipment is unpacked by someone unaware of cleaning protocol, lack of access to cleaning supplies	Risk of transmission of infection to the researcher *	2	6	7	84

*ABPM = ambulatory blood pressure monitoring, n/a = not applicable.* *Risk was deemed only to be to the researcher as the equipment is quarantined for a minimum of 72 hours upon return, before dispatch to another study participant.

***Risk priority numbers.*** The total RPN across all 42 sub-steps and their associated failure modes was 248. The lowest score assigned to any sub-step and associated failure modes was 0 (with 16 sub-steps scoring 0) and the highest was 84. The majority of the sub-steps and associated failure modes were deemed very low risk and scored 10 or less (36, 86%). We identified 5 low-to-moderate risk sub-steps (12%) and one moderate-to-high risk sub-step. We prioritised these two groups for the proactive identification of risk-reduction strategies for mitigating failure of the remote ABPM process. Of note, there was only 1 sub-step (arranging courier delivery and return of the ABPM) that was unique to the remote setting of ABPM; this was identified low-to-moderate risk. All other sub-steps were inherent to the ABPM process itself, whether performed face-to-face or remotely. The sub-steps with failure modes that were scored as low-to-moderate risk (RPN 11–50) or moderate-to-high risk (RPN greater than 50) are reported in Table 3.

***Strategies for risk reduction in the remote ABPM process.*** The FMEA panel developed strategies for proactive risk reduction to prevent failure to the remote ABPM process. Examples include creating a checklist of eligibility criteria against which participants should be re-screened when booking their remote ABPM fitting appointment and a checklist for the information required from participants to accurately configure the monitor to their schedule. Other strategies included refining written instructions and photographs regarding how to position the monitor tubing for the 24-hour period of wear with the panel patient and public representative. For the highest scoring sub-step and failure mode (cleaning monitoring equipment on its return to the research centre), strategies developed included ensuring the equipment was returned personally to those staff trained in the study procedures to avoid the parcel being opened by non-trained staff, adding cleaning instructions to the ABPM download instructions as the first step in this process, and a clear process for escalation in the event of diminishing or absent cleaning supplies.

### Assessment of successful ambulatory blood pressure monitoring episodes

**Figure 2.**
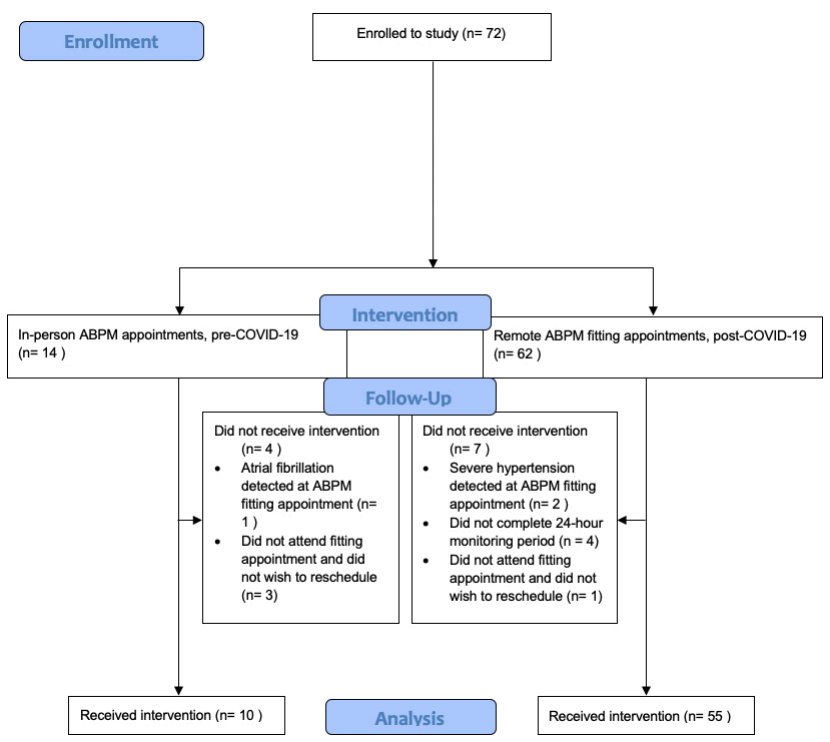
Participant flow diagram.

Between 17 January 2020 and 10 March 2020, prior to the first COVID-19 lockdown in the UK, 14 face-to-face ABPM appointments were arranged and 10 (71%) were completed; three (21%) participants did not attend their scheduled fitting appointment and one (2%) was not undertaken due to the detection of atrial fibrillation at their fitting appointment, warranting same-day medical referral ( Figure 2). Following resumption of research activity with easing of COVID-19 restrictions, 61 remote-ABPM fitting appointments were arranged between 9 December 2020 and 16 August 2021 and 54 (89%) were completed. Two (3%) of these participants were not able to proceed to ABPM due to the detection of severe hypertension at their remote fitting appointments (warranting same-day medical referral); one (2%) participant did not attend their remote fitting appointment and did not wish to reschedule and 4 (6%) participants did not complete their 24-hour monitoring period. Two (3%) participants switched their ABPM monitor off at night-time but complied with the day-time monitoring. Two (3%) fitting appointments required rescheduling due to issues with courier delivery of the monitors. All monitors were safely returned to the research centre after completion of ABPM with no loss of data.

Of the 10 ABPM episodes performed via face-to-face fitting and removal, all were successful and obtained sufficient data (defined by the National Institute for Health and Care Excellence as ≥14 daytime measurements ^5^) for diagnostic analysis. Mean age of these participants was 59 years and 80% were male. Similarly, of the 55 completed ABPMs performed with remote fitting, all were successful and obtained sufficient data for diagnostic analysis. Mean age of these participants was 50 years and 57% were male. For the 10 ABPM episodes with face-to-face fitting, there were 402 attempted blood pressure measurements, of which 361 (89%) were successful. Across the 55 participants who underwent remote fitting appointments, there were 2516 attempted measurements, of which 2,214 (88%) were successful. There was no significant difference between the mean error rate per participant between the face-to-face and remote ABPM cohorts (mean error rates 0.100 [SD 0.009] and 0.143 [SD 0.132] respectively, 95% confidence interval for the difference being -0.125 to 0.045 and two-tailed P value 0.353).

### Safety procedures

All ECGs were reviewed and manually interpreted by a GP on return of the tablet computers for any instances of atrial fibrillation missed by the automated interpretation of the ECG via the Kardia app; no missed instances of atrial fibrillation were detected. Similarly, all ABPM reports were reviewed by two research clinicians (a research physiotherapist and a GP) for any instances of severely elevated blood pressure not detected at the fitting appointment and none were detected.

## Discussion

### Summary of results

We compared the performance of ABPM when delivered via face-to-face clinic appointments, versus when delivered remotely using telemedicine in a research study setting. We observed no statistically significant or clinically important difference in the performance of the monitoring between the two settings and demonstrated that monitors can be reliably and safely configured, fitted and removed for return using remote telemedicine consulting. Our safety procedures at the fitting appointments were effective at detecting one person with atrial fibrillation and two with severely elevated blood pressure. Non-attendance rates for ABPM were markedly higher in the face-to-face monitoring group (three participants, 21%) than the remote monitoring group (seven participants, 12%). Of the seven participants in the remote monitoring group who did not attend, six rearranged and attended a rescheduled appointment. We observed a greater number of blood pressure measurements per monitoring period in the remote ABPM group than the face-to-face group, likely owing to a greater flexibility in appointment times following the adoption of the remote process. When performing face-to-face ABPM appointments, appointment times were limited by the schedule of pre-booked clinic rooms, and to ensure adequate monitoring periods for all participants attending each clinic, we scheduled participants two appointments exactly 24 hours apart. However, with the move to remote monitoring, we were able to offer participants greater flexibility in appointment time.

We performed a Failure Modes and Effects Analysis of the process of remotely delivered ABPM and observed a single high-risk step, which related to the cleaning of equipment in the context of a global COVID-19 pandemic, and did not pertain to the remote nature of the process.

Overall, we observed very low RPNs when performing the FMEA for remote ABPM. There are two potential contributing factors to these low-risk scores. Firstly, ABPM is a safe and non-invasive clinical test delivered routinely in clinical care and known to be associated with minimal risk. Secondly, the study processes had already been designed to minimise risk to participants and the study, such as careful screening for eligibility for study inclusion and suitability for ABPM at baseline enrolment, with further eligibility checks at the point of arranging and fitting the ABPM.

### Strengths and limitations

We undertook a mixed-methods approach to evaluating the process and performance of remote ABPM. We performed a quantitative analysis of the ABPM data obtained through face-to-face ABPM fittings and remote ABPM fittings, using all data available from both groups at the time of performing the analysis. We performed an in-depth risk analysis of the remote ABPM process, using FMEA and with broad representation on the FMEA panel.

We were not able to calculate our sample size *a priori* to ensure it was powered to detect a significant difference in the proportion of successful episodes of ABPM or attempted measurements during each ABPM episode. This was due to the face-to-face cohort size being defined by the short time period in which we were able to recruit and follow up participants before suspension of research activity due to the first wave of COVID-19 in the UK. Our comparative analysis of the mean error rate between the two cohorts is therefore vulnerable to a type II error. However, the width and magnitude of the calculated 95% confidence interval is small. We elected not to resume face-to-face ABPM appointments following the gradual lifting of COVID-19 restrictions in the UK, as we observed a number of benefits to the remote appointment offering, alongside the quality of remote ABPM appearing equivalent when compared to ABPM set up via face-to-face appointments. These observed benefits included greater patient interest in enrolling to the study, which translated to higher recruitment rates, lower rates of ‘no shows’ for booked ABPM appointments and removal of the barrier of travel and parking at healthcare centres for face-to-face appointments. Furthermore, pressures on primary care clinic room space in healthcare facilities are a major challenge in the UK at the present time and securing clinic space at times and locations convenient to participants was a major challenge ^15^.

We primarily assessed the success of each ABPM episode against NICE Guidelines’ recommendation of having at least 14 daytime measurements available from ABPM, due to the study setting being in the UK ^6^. The NICE Guidelines for the diagnosis and management of hypertension do not make any recommendations for diagnosing blood pressure using night-time measurements. However, internationally, the full 24-hour period of monitoring is considered when making a diagnosis of hypertension ^7, 16^. We therefore also assessed the rate of successful BP measurements within the 24-hour period of ABPM for both groups of participants. In the full analysis for the over-arching diagnostic accuracy study, from which this data has been obtained, we will also assess the rate of nocturnal hypertension and 24-hour hypertension, using international guidelines ^1^.

The population in this study may not be representative of typical patients who are offered ABPM in the real-world clinical setting and further research evaluating this remote process in other settings is recommended.

### Comparison with existing literature

We consider the approach to delivering and evaluating remote ABPM as described in this study to be novel. Our demonstration of the reliability and safety of remote ABPM may help primary care and hypertension clinicians and researchers consider whether existing services can be adapted to resume a resilient delivery of this important component of hypertension diagnostics and care.

Several other researchers have used FMEA to analyse the safety of healthcare environments that have been impacted by COVID-19 ^17– 19^. However, we have not identified any studies that have used FMEA to evaluate the adaptation of a specific medical procedure with the aim of reducing risk of transmission of COVID-19, such as this present study. We found FMEA a useful tool for this purpose; the systematic approach helped identify the risks associated with the specific adaptation of ABPM to a remote service.

### Implications for research and clinical practice

In 2018, the WHO Regional Office for Europe launched a roadmap for the digitalisation of national health systems and in 2019, the NHS Long Term Plan for England outlined how digitally-enabled outpatient and primary care will become ‘mainstream’ throughout the NHS ^20^. COVID-19 has necessitated an accelerated digitalisation of healthcare services and our findings support ABPM being one such service that may be digitally-enabled and offered remotely.

ABPM is a safe and routine procedure in every-day clinical care, and we have highlighted the key potential failure points that could occur when delivering this remotely which will likely be of interest to clinical and research services. However, researchers would need to consider the applicability of the risks assessed here to any other research and clinical settings in which remote ABPM is proposed.

The remote ABPM package in this study included a CE-marked and FDA approved mobile ECG device to screen for atrial fibrillation as part of our eligibility checks given we stated, a priori, that people with atrial fibrillation would be excluded from this study. This decision was made due to the overarching clinical study being one of diagnostic accuracy, and the reliability of automated blood pressure measurements in the context of atrial fibrillation has been debated ^21^. It is possible however, that a clinical service offering remote ABPM may not require the inclusion of a mobile ECG device if an ambulatory blood pressure monitor with proven reliability and validity in the context of atrial fibrillation could be deployed. A previous systematic review and meta-analysis investigating the reliability of automated blood pressure machines in the context of atrial fibrillation included six studies of four ambulatory blood pressure meachines; this found that ambulatory measurements of systolic blood pressure performed with two specific devices were comparable to readings with mercury sphygmomanometers ^22^. Interestingly, the authors also found no evidence that ambulatory blood pressure monitors which are able to detect atrial fibrillation are any more accurate at blood pressure measurements in the context of atrial fibrillation than monitors without this function.

The costs of adapting services to offer remote ABPM need consideration in both research and clinical settings; associated costs with the remote delivery include the use of mobile ECG devices and courier usage. In addition, for this study we utilised study-owned tablet computers for video calling and ECG interpretation, to promote inclusivity of participation and remove requirements on participants to download applications to their personal devices. As mobile device ownership and digital literacy become ubiquitous among the communities in need of this service, provision of this equipment may become unnecessary. Similarly, as described above, if this remote method of ABPM measurement were to be implemented at scale in clinical care, outside of the context of a diagnostic accuracy study, provision of an ECG device for the screening of atrial fibrillation may not be required. The costs associated with remote delivery of ABPM may be offset by the costs of a clinic room and societal costs to patients and participants in travelling to face-to-face clinic appointments. However, a full economic evaluation would be required to understand this in greater detail and to inform decisions over adaptation of clinical services. Such an economic evaluation may also include home blood pressure monitoring as an additional comparator ^23^.

## Conclusion

We have demonstrated that ABPM can be safely and appropriately provided in the community remotely and without face-to-face contact, using video technology for remote fitting appointments, alongside courier services for delivery of equipment to participants. This remote service has been instrumental in resuming research activity whilst mitigating the risk of COVID-19 transmission between participants and researchers. The COVID-19 pandemic has presented an opportunity for reconfiguring services, in this case to deliver a more accessible service for patients and one that is resilient to disruptions in usual care. Looking to the future, ABPM could be one service that is digitally enabled in both primary and secondary care.

## Data availability

Oxford Research Archive for Data: Remote versus face-to-face ambulatory blood pressure monitoring: de-identified blood pressure data from 65 participants of the Screening for Hypertension in the Inpatient Environment (SHINE) study, https://doi.org/10.5287/bodleian:qa9ZZmdOJ).

Data are available under the terms of the Creative Commons Attribution 4.0 International license (CC-BY 4.0). 
