# Supplementary material for: Ambulatory blood pressure monitoring using telemedicine: proof-of-concept cohort and failure modes and effects analyses
Source: Wellcome Open Res. 2022 Aug 24;7:39. Originally published 2022 Feb 3. [Version 3] doi: 10.12688/wellcomeopenres.17537.3 (PMC9411972; doi:10.12688/wellcomeopenres.17537.3)
Supplement: Peer review history

# Peer review: Reviewer response for version 3

**DOI:** 10.21956/wellcomeopenres.20271.r52146

**Published:** 2022-08-25

**Authors:** Christian Delles

**Affiliations:** 1 School of Cardiovascular and Metabolic Health, University of Glasgow, Glasgow, UK

Thank you for providing further information. I am happy with the changes that you made.

Is the work clearly and accurately presented and does it cite the current literature?

Yes

If applicable, is the statistical analysis and its interpretation appropriate?

Partly

Are all the source data underlying the results available to ensure full reproducibility?

No

Is the study design appropriate and is the work technically sound?

Yes

Are the conclusions drawn adequately supported by the results?

Partly

Are sufficient details of methods and analysis provided to allow replication by others?

Partly

Reviewer Expertise:

Cardiovascular diseases

I confirm that I have read this submission and believe that I have an appropriate level of expertise to confirm that it is of an acceptable scientific standard.

# Peer review: Reviewer response for version 2

**DOI:** 10.21956/wellcomeopenres.19705.r51412

**Published:** 2022-07-19

**Authors:** Christian Delles

**Affiliations:** 1 School of Cardiovascular and Metabolic Health, University of Glasgow, Glasgow, UK

Laura Armitage and colleagues describe their experience with a remote setup for ambulatory blood pressure monitoring. They share their experience with others who may also have made adaptations to their hypertension service.

 I enjoyed reading the paper, but it is not necessarily the most concise paper I have ever seen. For example, within just three columns spanning pages 3 and 4, the authors mention three times that they adhered to the BIHS protocol. The abstract is not a particularly easy read. Still, I appreciate that rounds of revisions don’t always help to improve a paper and I will not request that the authors shorten their paper.

 I have, however, a number of other comments.

1. First of all I should say that I fully agree with Dr Omboni’s comment of definition of a valid ABPM. The introduction of the paper praises ABPM compared to HBPM, but then defining success based on daytime readings alone is counterintuitive – even if this is what NICE says. I note the authors’ response but still wonder when we will see the night time data. Will you really get back to this small group in a future full paper and report the here missing data for this group?
2. I agree that the data are convincing in terms of feasibility and performance but I am not sure if the readings are accurate. I appreciate that even repeated ABPM in a face-to-face setting will never be exactly the same but just from the fact that there were successful readings once cannot conclude that the measurements were indeed precise. This would require further study.
3. I do not fully understand when (how many weeks/months later?) after discharge from hospital these ABPMs were done. Please specify this and provide data.
4. Please clarify data in Table 1. Is the definition of severity at download also “>=160 or >=105” like in other rows or is it here “>=160/105” in the sense of “>=160 and >=105”?
5. I am really missing clinical data. The authors mention on page 11 that the “population in this study may not be representative of typical patients who are offered ABPM in the real-world clinical setting…”. One way to help the reader comparing the present patients with their own patients would be to provide demographic and clinical characteristics including blood pressure readings and medication. I have seen a brief mention of age and gender but not any blood pressure readings or clinical characteristics. These would make a nice table.
6. I am a bit unsure how long the “telemedicine” sessions took. Could you specify this please? I appreciate that this is not the time for a detailed cost effectiveness analysis but could you provide very simple data that could help readers to speak to their own services if they want to do something similar? What were your courier costs? How much additional time did you need for packing/shipping/unpacking the equipment? How much time did remote sessions take (see above)?
7. I note that cleaning of the equipment was the single high-risk step in the FMEA. I appreciate this. But is this a real risk or is it a perceived risk? I am not aware that handling blood pressure equipment was a major driver of the COVID-19 pandemic. A critical discussion of this topic (with the benefit of hindsight) is missing. Did you use disposable cuffs? Or standard cuffs and washed them? Has cleaning of cuffs changed with onset of the pandemic compared to your previous practice? I would assume that you didn’t send ABPM devices to patients how had COVID-19 or symptoms of COVID-19 and this was probably one of the screening questions? So the risk should be really low and I wonder why this was such a concern.
8. Finally, I appreciate that the remote service offers greater flexibility but assume this is still within regular working hours? I was surprised to read that you offered F2F appointments only with an exact 24-hours interval. Surely it would be possible to switch a device off and return it later, e.g. after a weekend or in the evening? So maybe this study also offers us learning points to be taken into account for conventional services as well.

Minor issues:

1. On page 3, right column, it should read “British and Irish Hypertension (not: Heart) Society” like elsewhere in the text.
2. I can’t follow the maths on page 9, right column. If one out of 14 was not undertaken due to AF that would be 1/14=0.07, i.e. 7% rather than 2%?

Is the work clearly and accurately presented and does it cite the current literature?

Yes

If applicable, is the statistical analysis and its interpretation appropriate?

Partly

Are all the source data underlying the results available to ensure full reproducibility?

No

Is the study design appropriate and is the work technically sound?

Yes

Are the conclusions drawn adequately supported by the results?

Partly

Are sufficient details of methods and analysis provided to allow replication by others?

Partly

Reviewer Expertise:

Cardiovascular diseases

I confirm that I have read this submission and believe that I have an appropriate level of expertise to confirm that it is of an acceptable scientific standard, however I have significant reservations, as outlined above.

# Response

**Published:** 2022-08-18

**Authors:** Laura Armitage

**Affiliations:** University of Oxford, UK

Laura Armitage and colleagues describe their experience with a remote setup for ambulatory blood pressure monitoring. They share their experience with others who may also have made adaptations to their hypertension service.

 I enjoyed reading the paper, but it is not necessarily the most concise paper I have ever seen. For example, within just three columns spanning pages 3 and 4, the authors mention three times that they adhered to the BIHS protocol. The abstract is not a particularly easy read. Still, I appreciate that rounds of revisions don’t always help to improve a paper and I will not request that the authors shorten their paper.

 I have, however, a number of other comments.

1. First of all I should say that I fully agree with Dr Omboni’s comment of definition of a valid ABPM. The introduction of the paper praises ABPM compared to HBPM, but then defining success based on daytime readings alone is counterintuitive – even if this is what NICE says. I note the authors’ response but still wonder when we will see the night time data. Will you really get back to this small group in a future full paper and report the here missing data for this group?

*Author response: Thank you for this comment. The night-time BP data are available with all data in the linked data repository. We have committed, a priori, in the SHINE study protocol to assess the rate of nocturnal hypertension and 24-hour hypertension using international guidelines and have added a statement about this commitment to the present article. We have another article in print at present, highlighting the importance of assessing night-time BP as we agree that the present NICE guidelines are limited. Recent evidence points to the need for 24-hour, including night-time, BP assessments, in line with hypertension diagnostic guidelines globally.*

1. I agree that the data are convincing in terms of feasibility and performance but I am not sure if the readings are accurate. I appreciate that even repeated ABPM in a face-to-face setting will never be exactly the same but just from the fact that there were successful readings once cannot conclude that the measurements were indeed precise. This would require further study.

*Author response: Thank you for this comment. Our conclusion is that ABPM can be delivered safely and appropriately in the community remotely and we have been careful around our use of language, not asserting any certainty over accuracy. We have previously discussed as a group, how level of accuracy could be determined, and this would be extremely difficult to study because, as you say, ABPM varies day-to-day and so it wouldn’t be adequate to ask the same individuals to wear a monitor fitted remotely during one 24-hour period and then wear a monitor fitted face-to-face for another 24-hour period. The way around this could be for individuals to wear one monitor on each arm for the same 24-hour period, with one fitted remotely and one face-to-face, but problems with this could include additional discomfort and inconvenience to participants and difficulty going about every-day life whilst wearing the monitors, and possibly contamination between the two settings given participants would have the same instructions repeated at each appointment (remote and face-to-face) - so whichever came second may be optimised in terms of fitting and therefore accuracy. *

1. I do not fully understand when (how many weeks/months later?) after discharge from hospital these ABPMs were done. Please specify this and provide data.

*Author response: ABPMs were conducted between 1 and 6 months post-hospital discharge. The mean time between discharge and ABPM fitting was 54 days in the face-to-face ABPM cohort and 57 days in the remote ABPM cohort. This detail has been added in the results section under the sub-header ‘Assessment of successful ambulatory blood pressure monitoring episodes’.      *                                                                                                                                                                                         

  

1. Please clarify data in Table 1. Is the definition of severity at download also “>=160 or >=105” like in other rows or is it here “>=160/105” in the sense of “>=160 and >=105”?

*Author response: Thank you, we have now clarified this.*

  

1. I am really missing clinical data. The authors mention on page 11 that the “population in this study may not be representative of typical patients who are offered ABPM in the real-world clinical setting…”. One way to help the reader comparing the present patients with their own patients would be to provide demographic and clinical characteristics including blood pressure readings and medication. I have seen a brief mention of age and gender but not any blood pressure readings or clinical characteristics. These would make a nice table.

*Author response: The blood pressure data are available in the linked data repository as required by Wellcome Open Research. The aim of this study was to establish proof-of-concept and feasibility of a remote ABPM procedure and so it is out-with the scope of this present manuscript to analyse the results of the blood pressure data. Once a full dataset for the SHINE study is achieved, we will fulfil all of the study objectives in the published protocol, which include an analysis of the diagnostic blood pressure data, including in the context of patient clinical characteristics. However, we have now provided some further participant characteristics in this paper in Table 5. *

1. I am a bit unsure how long the “telemedicine” sessions took. Could you specify this please? I appreciate that this is not the time for a detailed cost effectiveness analysis but could you provide very simple data that could help readers to speak to their own services if they want to do something similar? What were your courier costs? How much additional time did you need for packing/shipping/unpacking the equipment? How much time did remote sessions take (see above)?

*Author response: Thank you, we have added this detail to the Discussion under the 4 ^th^ paragraph of the ‘Implications for research and clinical practice section’.*

  

1. I note that cleaning of the equipment was the single high-risk step in the FMEA. I appreciate this. But is this a real risk or is it a perceived risk? I am not aware that handling blood pressure equipment was a major driver of the COVID-19 pandemic. A critical discussion of this topic (with the benefit of hindsight) is missing. Did you use disposable cuffs? Or standard cuffs and washed them? Has cleaning of cuffs changed with onset of the pandemic compared to your previous practice? I would assume that you didn’t send ABPM devices to patients who had COVID-19 or symptoms of COVID-19 and this was probably one of the screening questions? So the risk should be really low and I wonder why this was such a concern.

*Author response: Thank you for your comments on this. We used standard cuffs and washed them. The cleaning of the cuffs did not change with the onset of the pandemic compared to previous practice, nor did the cleaning of the monitors; however, it was perceived that if cleaning was not adhered to the risk of infection transmission could be significant but as we have stated, this is irrespective of whether the ABPM appointments are conducted face-to-face or remotely. Around the time of carrying out the Failure Modes and Effects Analysis for this work, the significance of surface contamination and contact transmission was being debated within the literature and we have now added a critical discussion of this point to our article as you have suggested.*


*  *

1. Finally, I appreciate that the remote service offers greater flexibility but assume this is still within regular working hours? I was surprised to read that you offered F2F appointments only with an exact 24-hours interval. Surely it would be possible to switch a device off and return it later, e.g. after a weekend or in the evening? So maybe this study also offers us learning points to be taken into account for conventional services as well.

*Author response: Thank you for this comment. The majority of the time, the remote service was within regular working hours. Unfortunately it wasn’t possible to offer such flexibility with face-to-face appointments with regard to return of the monitors due to severe constraints on clinic room availability in GP surgeries, which is a national issue; we therefore needed to block-book clinic appointments and the most effective way we found to do this, to suit the majority of participants, ensure room availability and manage the human resource required to travel to clinics was to block book a clinic room for a session on 2 consecutive days, and therefore book participants into two appointments 24-hours apart. We have briefly added some further discussion of this point.*

Minor issues:

1. On page 3, right column, it should read “British and Irish Hypertension (not: Heart) Society” like elsewhere in the text.

*Author response: Thank you, this is now amended.*

  

1. I can’t follow the maths on page 9, right column. If one out of 14 was not undertaken due to AF that would be 1/14=0.07, i.e. 7% rather than 2%?

*Author response: Thank you, this has been corrected*

# Peer review: Reviewer response for version 2

**DOI:** 10.21956/wellcomeopenres.19705.r49793

**Published:** 2022-04-07

**Authors:** Stefano Omboni

**Affiliations:** 1 Clinical Research Unit, Italian Institute of Telemedicine, Varese, Italy; 2 Department of Cardiology, First Moscow State Medical University, Moscow, Russian Federation

The authors have thoroughly and adequately responded to the remarks I previously raised. I have no further comments.

Is the work clearly and accurately presented and does it cite the current literature?

Yes

If applicable, is the statistical analysis and its interpretation appropriate?

Yes

Are all the source data underlying the results available to ensure full reproducibility?

Yes

Is the study design appropriate and is the work technically sound?

Yes

Are the conclusions drawn adequately supported by the results?

Partly

Are sufficient details of methods and analysis provided to allow replication by others?

Yes

Reviewer Expertise:

telemedicine; ambulatory blood pressure monitoring; hypertension

I confirm that I have read this submission and believe that I have an appropriate level of expertise to confirm that it is of an acceptable scientific standard.

# Peer review: Reviewer response for version 1

**DOI:** 10.21956/wellcomeopenres.19391.r48645

**Published:** 2022-02-17

**Authors:** Stefano Omboni

**Affiliations:** 1 Clinical Research Unit, Italian Institute of Telemedicine, Varese, Italy; 2 Department of Cardiology, First Moscow State Medical University, Moscow, Russian Federation

In this paper, the authors demonstrate that ABPM can be safely and appropriately provided in the community remotely and without face-to-face contact using telemedicine. The study is novel in its kind, interesting, and well presented, and it confirms what is documented in other studies in different settings (e.g., pharmacies).

 The authors set to use the NICE criteria for evaluating the ABPM quality ("When using ABPM to confirm a diagnosis of hypertension, ensure that at least two measurements per hour are taken during the person's usual waking hours (for example, between 08:00 and 22:00). Use the average value of at least 14 measurements taken during the person's usual waking hours to confirm a diagnosis of hypertension.”). However, these criteria are questionable because they are too loose compared to International ones. The decision to perform a 24-hour ABPM and then limit the diagnosis to daytime hours is also questionable. Since a significant proportion of patients may have night-time hypertension (particularly older people and those treated with antihypertensive medication), including recordings deemed valid only for the waking hours, but potentially invalid for night-time hours and excluding the night-time period from the diagnostic assessment may be a significant source of diagnostic inaccuracy. The authors must discuss this aspect as a study limitation. In the future, I would recommend applying more strict quality criteria to ensure high diagnostic accuracy.

 Did any patient have missing BP readings during the night-time? The author should indicate whether readings were available for the whole 24 hours for all recordings.

 The use of a mobile ECG device shipped to the user and the ABPM monitor might be a complication in the routine workout of the service. Current ABP monitors can detect the occurrence of arrhythmias (also atrial fibrillation in some cases) as well as smartwatches that are available to many young people. The authors may discuss this aspect in the "Implications for research and clinical practice" section.

 A significant limitation of this study is the small sample size of the face-to-face cohort. The authors discuss this aspect and acknowledge this limitation. Nevertheless, this is a significant limitation that would deserve a more thorough discussion. For instance, did authors resume face-to-face appointments after the lockdown? Were they able to recruit more patients in that cohort after the lockdown or isolation? This is not mentioned on page 9 ("Assessment of successful ambulatory blood pressure monitoring episodes")

Is the work clearly and accurately presented and does it cite the current literature?

Yes

If applicable, is the statistical analysis and its interpretation appropriate?

Yes

Are all the source data underlying the results available to ensure full reproducibility?

Yes

Is the study design appropriate and is the work technically sound?

Yes

Are the conclusions drawn adequately supported by the results?

Partly

Are sufficient details of methods and analysis provided to allow replication by others?

Yes

Reviewer Expertise:

telemedicine; ambulatory blood pressure monitoring; hypertension

I confirm that I have read this submission and believe that I have an appropriate level of expertise to confirm that it is of an acceptable scientific standard, however I have significant reservations, as outlined above.

# Response

**Published:** 2022-03-20

**Authors:** Laura Armitage

**Affiliations:** University of Oxford, UK

**Outcome:** Approved with reservations

 Ambulatory blood pressure monitoring using telemedicine: proof-of0concept cohort and failure modes and effects analyses.

 In this paper, the authors demonstrate that ABPM can be safely and appropriately provided in the community remotely and without face-to-face contact using telemedicine. The study is novel in its kind, interesting, and well presented, and it confirms what is documented in other studies in different settings (e.g., pharmacies).

**Author response:**

 Thank you for this feedback

- *The authors set to use the NICE criteria for evaluating the ABPM quality ("When using ABPM to confirm a diagnosis of hypertension, ensure that at least two measurements per hour are taken during the person's usual waking hours (for example, between 08:00 and 22:00). Use the average value of at least 14 measurements taken during the person's usual waking hours to confirm a diagnosis of hypertension.”). However, these criteria are questionable because they are too loose compared to International ones. The decision to perform a 24-hour ABPM and then limit the diagnosis to daytime hours is also questionable. Since a significant proportion of patients may have night-time hypertension (particularly older people and those treated with antihypertensive medication), including recordings deemed valid only for the waking hours, but potentially invalid for night-time hours and excluding the night-time period from the diagnostic assessment may be a significant source of diagnostic inaccuracy. The authors must discuss this aspect as a study limitation. In the future, I would recommend applying more strict quality criteria to ensure high diagnostic accuracy.*
- **Response: **Thank you very much for this feedback. We acknowledge your concerns regarding the NICE diagnostic criteria for hypertension and in our study protocol for the diagnostic accuracy study ( https://bmjopen.bmj.com/content/9/12/e033792.long) we have planned, a priori, to perform additional analyses incorporating night-time measurements and using the European and American diagnostic thresholds for hypertension. We have added the following text regarding this to our discussion under the sub-header Strengths and limitations to the present manuscript: “We primarily assessed the success of each ABPM episode against NICE Guidelines’ recommendation of having at least 14 daytime measurements available from ABPM, due to the study setting being in the UK. ^6^ The NICE Guidelines for the diagnosis and management of hypertension do not make any recommendations for diagnosing blood pressure using night-time measurements. However, internationally, the full 24-hour period of monitoring is considered when making a diagnosis of hypertension. ^8,15^ We therefore also assessed the rate of successful BP measurements within the 24-hour period of ABPM for both groups of participants. In the full analysis for the over-arching diagnostic accuracy study, from which this data has been obtained, we will also assess the rate of nocturnal hypertension and 24-hour hypertension, using international guidelines. ^1^ “
- *Did any patient have missing BP readings during the night-time? The author should indicate whether readings were available for the whole 24 hours for all recordings.*
- **Response: **Thank you for this comment. In the pre-COVID, face-to-face ABMP group, none of the participants switched off their monitor during the night-time. In the post-COVID, remote ABPM group, two participants switched off their monitor during the night-time. We have added this detail to the manuscript under the Results sub-header ‘Assessment of successful ambulatory blood pressure monitoring episodes’. The error rate reported for both groups has been calculated using all blood pressure values measured during the full period of wear (both day-time and night-time values).
- *The use of a mobile ECG device shipped to the user and the ABPM monitor might be a complication in the routine workout of the service. Current ABP monitors can detect the occurrence of arrhythmias (also atrial fibrillation in some cases) as well as smartwatches that are available to many young people. The authors may discuss this aspect in the "Implications for research and clinical practice" section.*
- **Response:** Thank you, our reason for screening for atrial fibrillation using the ECG device was due to the debated reliability of automated blood pressure machines in the context of AF. The use of a monitor which has proven reliability and validity in the context of AF would be of most important to the diagnostic accuracy of the results. We note a recent review by Clark et al made an important distinction between ABPM monitors that can identify AF and those which are reliable in the context of AF. We have added some to the section ‘Implications for research and clinical practice’ as follows: “The remote ABPM package in this study included a CE-marked and FDA approved mobile ECG device to screen for atrial fibrillation as part of our eligibility checks given we stated, a priori, that people with atrial fibrillation would be excluded from this study. This decision was made due to the overarching clinical study being one of diagnostic accuracy, and the reliability of automated blood pressure measurements in the context of atrial fibrillation has been debated. ^20^ It is possible however, that a clinical service offering remote ABPM may not require the inclusion of a mobile ECG device if an ambulatory blood pressure monitor with proven reliability and validity in the context of atrial fibrillation could be deployed. A previous systematic review and meta-analysis investigating the reliability of automated blood pressure machines in the context of atrial fibrillation included six studies of four ambulatory blood pressure machines; this found that ambulatory measurements of systolic blood pressure performed with two specific devices were comparable to readings with mercury sphygmomanometers. ^18^ Interestingly, the authors also found no evidence that ambulatory blood pressure monitors which are able to detect atrial fibrillation are any more accurate at blood pressure measurements in the context of atrial fibrillation than monitors without this function. “
- *A significant limitation of this study is the small sample size of the face-to-face cohort. The authors discuss this aspect and acknowledge this limitation. Nevertheless, this is a significant limitation that would deserve a more thorough discussion. For instance, did authors resume face-to-face appointments after the lockdown? Were they able to recruit more patients in that cohort after the lockdown or isolation? This is not mentioned on page 9 ("Assessment of successful ambulatory blood pressure monitoring episodes")*
- **Response: **Thank you for this question. We did not resume face-to-face ABPM when COVID-19 restrictions started to lift in the UK. We have updated the manuscript with the following text under the Strengths and Limitations section: “We elected not to resume face-to-face ABPM appointments following the gradual lifting of COVID-19 restrictions in the UK, as we observed a number of benefits to the remote appointment offering, alongside the quality of remote ABPM appearing equitable when compared to ABPM when set up via face-to-face appointments. These observed benefits included greater patient interest in enrolling to the study, which translated to higher recruitment rates, lower rates of ‘no shows’ for booked ABPM appointments and removal of the barrier of travel and parking at healthcare centres for face-to-face appointments. Furthermore, pressures on primary care clinic room space in healthcare facilities are a major challenge in the UK at the present time and securing clinic space at times and locations convenient to participants was a major challenge. ^14^”
